# Analytical similarity as the primary determinant of monoclonal antibody development: toward evidence-efficient regulatory frameworks

**DOI:** 10.3389/fddev.2026.1801236

**Published:** 2026-04-15

**Authors:** Sarfaraz K. Niazi

**Affiliations:** University of Illinois, Chicago, IL, United States

**Keywords:** analytical similarity, biosimilars, comparative efficacy studies, global harmonization, monoclonal antibodies, new approach methodologies, nonclinical safety, regulatory science

## Abstract

Monoclonal antibodies (mAbs) constitute the most rapidly expanding and therapeutically impactful class of biological medicines, driven by their exceptional target specificity, modular engineering capabilities, predictable pharmacokinetics facilitated by neonatal Fc receptor (FcRn) recycling, and favorable safety profiles attributable to mechanism-based pharmacology. As foundational patents on blockbuster antibody therapeutics expire, biosimilar antibodies have become a vital mechanism to enhance global accessibility, improve healthcare sustainability, and promote market competition. Simultaneously, regulatory agencies worldwide are substantially restructuring development expectations to emphasize analytical precision, mechanistic understanding, human-relevant methodologies, and ethical nonclinical practices aligned with the 3Rs principles of replacement, reduction, and refinement. This comprehensive review analyzes the convergence of innovator and biosimilar antibody development toward an analytics-first comparability paradigm, mechanism-based safety assessment, and targeted clinical confirmation based on residual uncertainty rather than prescriptive requirements. It systematically incorporates regulatory positions from the United States Food and Drug Administration (FDA), European Medicines Agency (EMA), World Health Organization (WHO), Japan’s Pharmaceuticals and Medical Devices Agency (PMDA), Health Canada, and the International Council for Harmonisation (ICH), together with contemporary peer-reviewed research literature. Focus is given to the FDA Modernization Act 2.0, which abolished statutory mandates for animal testing, and recent FDA draft guidance—which are provisional and open to revision pending finalization—addressing streamlined nonclinical safety studies for monospecific antibodies and science-based approaches to waiving comparative clinical efficacy studies (CES). The scientific boundaries distinguishing products amenable to streamlined development from those requiring tailored approaches are delineated, including the exclusion of polyclonal antibody preparations from biosimilar frameworks and the analytical complexities associated with conjugated antibodies and multispecific constructs. Important limitations of analytics-first approaches are also addressed, including scenarios involving heightened immunogenicity risk, novel targets with limited clinical experience, or insufficiently validated pharmacodynamic markers. These developments are examined alongside counterarguments and residual areas of regulatory skepticism. While the trajectory of regulatory reform appears to favor evidence-efficient, science-driven models for antibody development, this review explicitly distinguishes between established regulatory consensus, emerging draft guidance positions, and the author’s scholarly interpretation, recognizing that the pace and scope of regulatory change remain subject to ongoing deliberation.

## 1 Introduction

### 1.1 Central thesis and scope

This review advances a central thesis: that the progressive removal or de-emphasis of legacy regulatory requirements—such as routine animal toxicology studies and mandatory comparative clinical efficacy trials that may no longer provide decision-relevant information for well-characterized mAbs—may enable a more rational, evidence-based, and streamlined approach to biological drug development across both innovator and biosimilar pathways. The scientific foundation for this thesis rests on three principal pillars: (1) the platform predictability of mAbs arising from their conserved immunoglobulin scaffold; (2) the demonstrated sensitivity of modern analytical methods to detect clinically relevant molecular differences; and (3) the accumulated regulatory and clinical experience suggesting that comprehensive analytical and pharmacokinetic characterization can reliably predict clinical performance in many, though not all, contexts.

Unless otherwise specified, this review focuses on regulatory and scientific considerations relevant to biosimilar mAbs, with references to innovator antibody development included only where they inform comparability principles, lifecycle management, or regulatory precedent. The analytics-first framework discussed here is not universally applicable; limitations and boundary conditions are explicitly addressed in Section 8 and throughout the manuscript. It is important to note at the outset that this review represents the author’s scholarly interpretation of the evidence and regulatory landscape. Readers should distinguish between established regulatory requirements, emerging draft guidance proposals, and interpretive analysis presented herein.

### 1.2 The evolving therapeutic landscape

Monoclonal antibodies have fundamentally transformed therapeutic intervention across oncology, immunology, infectious diseases, neurology, and chronic inflammatory disorders, now accounting for a substantial and growing proportion of new biologic approvals worldwide and representing the dominant revenue segment within the global pharmaceutical market (Walsh, 2018; Kaplon et al., 2024). Their clinical success is rooted in a convergence of molecular and pharmacological factors: exquisite target specificity arising from complementarity-determining region (CDR) diversity, tunable effector functions mediated by the crystallizable fragment (Fc) region, predictable pharmacokinetics governed by FcRn recycling pathways, and the ability to engineer modular molecular architectures including bispecific, multispecific, and Fc-modified variants (Beck et al., 2010; Strohl, 2015; Carter and Lazar, 2018).

Historically, however, antibody development was constrained by regulatory paradigms inherited from small-molecule pharmacology, including extensive animal toxicology testing protocols and large comparative clinical efficacy trials designed to detect pharmacological differences rather than confirm molecular similarity. Over time, accumulated scientific evidence has suggested that these paradigms were frequently misaligned with antibody biology and the principles underlying biosimilar development. Unlike small molecules, which may exhibit off-target chemical toxicity through promiscuous receptor binding or reactive metabolite formation, mAbs rarely demonstrate unpredictable toxicity; instead, their adverse effects typically arise from exaggerated or unintended pharmacological engagement of the target pathway or from immunogenicity, both of which are largely predictable from mechanism-of-action analysis, target tissue expression patterns, and prior clinical experience with the reference product (Brennan et al., 2010; Ponce et al., 2009). However, the degree to which such predictability eliminates the need for empirical confirmation remains a subject of ongoing regulatory deliberation.

### 1.3 Regulatory evolution and scientific critique

Multiple independent analyses in the peer-reviewed literature have questioned whether clinical efficacy trials in biosimilar development are scientifically necessary when comprehensive analytical and pharmacokinetic similarity have been established. Schiestl et al. (2011) provided early evidence that analytical and functional assays may exhibit greater discriminatory power than clinical endpoints, which are inherently confounded by disease heterogeneity, background therapies, inter-patient variability, and placebo responses. Kurki et al. (2017) extended these observations in their analysis of European regulatory experience with biosimilar mAbs. Webster et al. (2019) further evaluated the role of pharmacovigilance data in supporting reduced clinical requirements for well-characterized biosimilars. Additionally, Niazi (2022a); Niazi (2025a) has argued that clinical efficacy trials are often statistically ill-suited to resolve residual uncertainty once analytical and pharmacokinetic similarity are established, contributing to broader discussions about evidence-efficient development pathways. It should be noted, however, that some regulatory scientists and industry stakeholders maintain that comparative clinical efficacy studies provide an essential safety net, particularly for products with limited post-marketing experience or those entering new therapeutic indications (Christl et al., 2017; Grampp and Ramanan, 2019). Barbier et al. (2020), in a systematic review of biosimilar switching studies, noted that while clinical outcomes were generally comparable, the evidentiary framework for confident CES waiver requires further strengthening through real-world evidence accumulation.

In response to this accumulating evidence base, regulatory agencies have increasingly embraced risk-based, evidence-efficient frameworks that emphasize analytical characterization, mechanistic understanding, and selective clinical confirmation predicated on residual uncertainty rather than prescriptive requirements (FDA, 2015; EMA, 2012; WHO, 2022). This evolution is reflected in EMA's pioneering biosimilar monoclonal antibody guideline (EMA, 2012), WHO's stepwise biosimilar evaluation framework (WHO, 2022), and most recently, FDA's 2025 draft guidances on streamlined nonclinical safety studies for monospecific antibodies and on science-based approaches to assessing the need for comparative clinical efficacy studies (FDA, 2025a; FDA, 2025b). It should be emphasized that these FDA draft guidances remain provisional pending public comment and finalization and represent current regulatory thinking rather than established policy. (European Medicines Agency, 2009; Health Canada, 2021; International Council for Harmonisation, 2005; International Council for Harmonisation, 2008; International Council for Harmonisation, 2009; International Council for Harmonisation, 2012; International Council for Harmonisation, 2019; U.S. Food and Drug Administration, 2011; U.S. Food and Drug Administration, 2022).

### 1.4 Current regulatory status versus emerging policy trends

To ensure clarity regarding the regulatory landscape discussed throughout this review, it is essential to distinguish among three categories of regulatory information:

Established regulatory requirements: These include binding guidelines and regulations that currently govern biosimilar development. Key examples include ICH S6(R1) on preclinical safety evaluation of biotechnology-derived pharmaceuticals (ICH, 2011), EMA’s Guideline on Similar Biological Medicinal Products Containing Monoclonal Antibodies (EMA, 2012; updated 2022), WHO’s Guidelines on Evaluation of Biosimilar Monoclonal Antibodies (WHO, 2022), and FDA’s 2015 guidance on demonstrating biosimilarity to a reference product (FDA, 2015). These documents represent finalized regulatory positions that sponsors are expected to follow.

Draft guidance proposals and emerging regulatory thinking: The FDA’s 2025 draft guidance on streamlined nonclinical safety studies for monospecific mAbs (FDA, 2025b) and updated recommendations for assessing the need for CES (FDA, 2025a) represent current agency thinking but remain subject to revision following public comment and finalization. The FDA’s 2025 Roadmap to Reducing Animal Testing in Preclinical Safety Studies (FDA, 2025c) similarly reflects directional intent rather than binding policy. Sponsors should not assume these draft positions will be adopted without modification.

Scholarly interpretation and analysis: The arguments presented in this review regarding the trajectory of regulatory reform, the sufficiency of analytical characterization to replace clinical efficacy trials in specific contexts, and the anticipated pace of global harmonization reflect the author’s interpretation of available evidence and regulatory signals. These interpretations are informed by peer-reviewed literature and regulatory documents but do not constitute regulatory guidance.

Throughout this review, language is calibrated to reflect these distinctions. Where draft guidance positions are discussed, they are explicitly identified as provisional. Where the author’s interpretive analysis extends beyond established regulatory consensus, this is noted.

### 1.5 Representative mAbs, biosimilars, and therapeutic proteins in clinical use

To provide a comprehensive overview of the current therapeutic landscape, Table 1 presents a representative listing of approved mAbs, their biosimilar counterparts, and key therapeutic proteins across major clinical applications.

**TABLE 1:** Representative approved monoclonal antibodies, biosimilars, and therapeutic proteins with their clinical applications. ADC, antibody-drug conjugate; AS, ankylosing spondylitis; CLL, chronic lymphocytic leukemia; CRC, colorectal cancer; CRS, cytokine release syndrome; DME, diabetic macular edema; GCA, giant cell arteritis; GPA, granulomatosis with polyangiitis; HCC, hepatocellular carcinoma; IBD, inflammatory bowel disease; JIA, juvenile idiopathic arthritis; MPA, microscopic polyangiitis; NHL, non-Hodgkin lymphoma; NSCLC, non-small cell lung cancer; PsA, psoriatic arthritis; RA, rheumatoid arthritis; RCC, renal cell carcinoma; UC, ulcerative colitis.

Product (Brand)	Type	Target	Therapeutic area	Clinical applications
*Representative innovator monoclonal antibodies*				
Adalimumab (Humira)	Humanized IgG1	TNF-α	Immunology	RA, psoriasis, Crohn’s, UC
Trastuzumab (Herceptin)	Humanized IgG1	HER2	Oncology	HER2+ breast cancer, gastric cancer
Bevacizumab (Avastin)	Humanized IgG1	VEGF-A	Oncology	CRC, lung, renal, cervical cancers
Rituximab (Rituxan)	Chimeric IgG1	CD20	Oncology/Immunol	NHL, CLL, RA
Pembrolizumab (Keytruda)	Humanized IgG4	PD-1	Oncology	Melanoma, NSCLC, multiple cancers
Nivolumab (Opdivo)	Human IgG4	PD-1	Oncology	Melanoma, RCC, HCC, NSCLC
Denosumab (Prolia/Xgeva)	Human IgG2	RANKL	Bone disease	Osteoporosis, bone metastases
Ustekinumab (Stelara)	Human IgG1	IL-12/IL-23	Immunology	Psoriasis, PsA, Crohn’s, UC
Dupilumab (Dupixent)	Human IgG4	IL-4Rα	Immunology	Atopic dermatitis, asthma
Daratumumab (Darzalex)	Human IgG1	CD38	Oncology	Multiple myeloma
Tocilizumab (Actemra)	Humanized IgG1	IL-6R	Immunology	RA, GCA, CRS
Pertuzumab (Perjeta)	Humanized IgG1	HER2	Oncology	HER2+ breast cancer (combination)
*Representative approved biosimilars*				
Adalimumab-atto (Amjevita)	Biosimilar	TNF-α	Immunology	All adalimumab indications
Trastuzumab-dkst (Ogivri)	Biosimilar	HER2	Oncology	HER2+ breast/gastric cancers
Bevacizumab-awwb (Mvasi)	Biosimilar	VEGF-A	Oncology	All bevacizumab indications
Rituximab-pvvr (Ruxience)	Biosimilar	CD20	Oncology/Immunol	NHL, CLL, RA, GPA, MPA
Denosumab (Wyost/Jubbonti)	Biosimilar	RANKL	Bone disease	Osteoporosis; bone metastases
Ustekinumab-hmny (Starjemza)	Biosimilar	IL-12/IL-23	Immunology	Psoriasis, PsA, IBD
Pertuzumab-dpzb (Poherdy)	Biosimilar	HER2	Oncology	HER2+ breast cancer
Tocilizumab-anoh (Avtozma)	Biosimilar	IL-6R	Immunology	RA, GCA, JIA
*Key Therapeutic proteins (non-antibody biologics)*				
Insulin glargine (Lantus)	Peptide hormone	Insulin receptor	Endocrinology	Type 1 and type 2 diabetes
Erythropoietin (Epogen)	Glycoprotein	EPO receptor	Hematology	Anemia (CKD, chemo-induced)
Filgrastim (Neupogen)	Recombinant G-CSF	G-CSF receptor	Hematology	Neutropenia
Etanercept (Enbrel)	Fc-fusion protein	TNF-α/β	Immunology	RA, psoriasis, AS
Aflibercept (Eylea)	Fc-fusion protein	VEGF-A/B, PlGF	Ophthalmology	Wet AMD, DME
Trastuzumab emtansine (Kadcyla)	ADC	HER2	Oncology	HER2+ metastatic breast cancer

## 2 Differentiating monoclonal antibodies from other therapeutic proteins

### 2.1 Structural and functional distinctiveness of the immunoglobulin scaffold

Although mAbs dominate the current biologics landscape, they represent only one category within the broader class of therapeutic proteins. A conserved immunoglobulin scaffold distinguishes mAbs—most commonly the IgG1 or IgG4 isotype—with a molecular weight of approximately 150 kDa, defined bivalent antigen-binding capacity, and Fc-mediated effector functions including antibody-dependent cellular cytotoxicity (ADCC), complement-dependent cytotoxicity (CDC), and antibody-dependent cellular phagocytosis (ADCP), as well as FcRn-mediated recycling that extends serum half-life (Wang et al., 2008; Dall’Acqua et al., 2002; Roopenian and Akilesh, 2007). Their pharmacokinetics are largely governed by FcRn-mediated salvage rather than by renal filtration or hepatic metabolism, thereby conferring extended half-lives of 10–30 days and relatively predictable exposure profiles across patient populations, with minimal influence from cytochrome P450 polymorphisms or drug-drug interactions (Mould and Meibohm, 2016).

**FIGURE 1:**
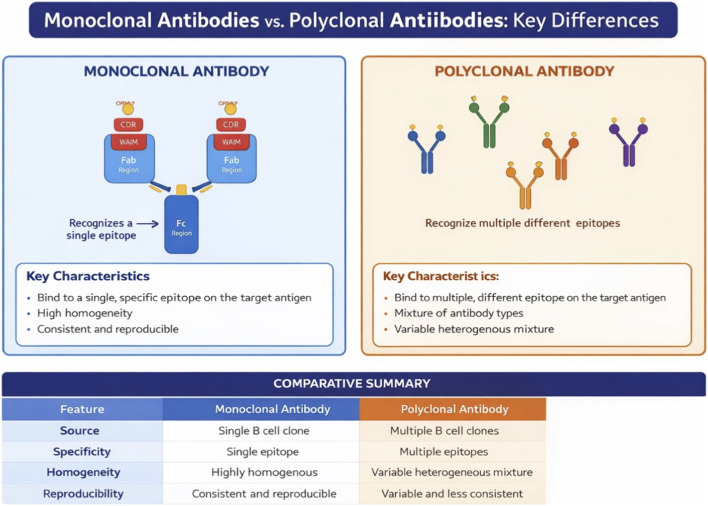
*Structural and functional comparison of monoclonal and polyclonal antibodies. Monoclonal antibodies (left) are homogeneous, derived from a single clone, and recognize a single epitope, making them amenable to biosimilar development through comprehensive analytical characterization. Polyclonal antibodies (right) are heterogeneous mixtures with multiple specificities, precluding biosimilar comparability assessment. This distinction is foundational to the analytics-first paradigm: the structural homogeneity and definability of mAbs enable the high-resolution analytical comparison upon which evidence-efficient development depends (see* Sections 6 and 9 *). Note: for expert audiences, this figure serves as an orienting reference; detailed structural analyses are presented in subsequent sections. Figures should be submitted in vector format (EPS or PDF) to ensure publication quality.*

By contrast, non-antibody therapeutic proteins—including peptide hormones, cytokines, enzymes, growth factors, and Fc-fusion proteins—exhibit far greater structural and functional diversity. Many lack FcRn-mediated recycling and are therefore rapidly cleared by renal filtration (for proteins below approximately 60 kDa), receptor-mediated endocytosis, or proteolytic degradation, rendering them more sensitive to formulation changes, aggregation, and manufacturing variability (Walsh, 2018; Leader et al., 2008) (Figure 1) (Table 1).

### 2.2 Regulatory implications of platform predictability

This biological distinction has critical regulatory consequences. Because mAbs share a common architectural platform and exhibit class-consistent pharmacokinetic and safety characteristics, regulators have developed antibody-specific guidance documents and relied on accumulated prior knowledge across the therapeutic class (FDA, 2015; EMA, 2012; ICH, 2011). The platform nature of antibodies has been emphasized in multiple regulatory science analyses as scientific support for streamlining biosimilar development (Weise et al., 2012; Kurki et al., 2017; Webster et al., 2019), with the argument that applying uniform, molecule-agnostic clinical requirements across all protein therapeutic classes may not account for fundamental differences in structure-function predictability and accumulated class-specific regulatory experience.

## 3 Post-translational modifications as a scientific boundary for streamlining

### 3.1 Glycosylation and Fc-region modifications in monoclonal antibodies

Post-translational modifications (PTMs) are intrinsic to the structure and function of most therapeutic proteins and represent a critical axis along which mAbs differ from many other biologics in terms of regulatory tractability. In mAbs, PTMs are predominantly localized to the Fc region, with N-linked glycosylation at asparagine 297 (Asn297) being the most prominent and functionally consequential modification. Fc glycosylation influences effector functions such as ADCC and complement activation. The functional consequences of glycan variability are relatively well characterized, and clinically acceptable ranges are often broad when anchored to the reference product’s historical manufacturing variability (Jefferis, 2009; Reusch and Tejada, 2015; Liu, 2015).

Extensive analytical and clinical experience accumulated across multiple biosimilar approvals has indicated that moderate differences in Fc glycosylation profiles do not necessarily translate into clinically meaningful differences in efficacy or safety, provided that functional activity as measured by validated bioassays remains comparable to the reference product within justified similarity ranges (Schiestl et al., 2011; Ferrara et al., 2011; Kozlowski et al., 2011). These conclusions assume that Fc-mediated effector functions are not the dominant determinants of clinical efficacy for the indication in question; situations involving narrow ADCC-dependent therapeutic windows or indications where effector function is critical to the mechanism of action may warrant additional scrutiny of glycosylation differences (Cartron et al., 2002).

### 3.2 PTM complexity in non-antibody proteins

By contrast, many non-antibody therapeutic proteins exhibit PTMs that are more heterogeneous, more functionally critical, or less tolerant to variation. Glycosylation patterns in erythropoiesis-stimulating agents directly influence receptor binding affinity, biological activity, and clearance rate; phosphorylation states may regulate enzymatic activity in enzyme replacement therapies; sulfation may be essential for specific ligand-receptor interactions (Elliott et al., 2003; Walsh and Jefferis, 2006; Berkowitz et al., 2012). In such cases, even minor PTM differences can lead to clinically meaningful changes in pharmacokinetics, potency, or immunogenicity, justifying a more conservative evidentiary approach that may require additional clinical studies to resolve residual uncertainty.

## 4 Foundations of animal testing requirements and their scientific reassessment

### 4.1 Historical regulatory context

Traditional nonclinical development of biologics has been guided by ICH M3(R2) and ICH S6(R1), which describe the role of animal toxicology in supporting clinical trials and marketing authorization for biotechnology-derived pharmaceuticals (ICH, 2009; ICH, 2011). While these guidelines already acknowledged significant limitations of animal models for antibodies lacking target cross-reactivity in available species, animal testing remained widely perceived as obligatory for regulatory compliance, often conducted without clear scientific justification or expectation of decision-relevant information (Chapman et al., 2012; Bussiere et al., 2009).

A decisive inflection point occurred with the passage of the FDA Modernization Act 2.0 in December 2022, which removed the statutory requirement for animal testing before human clinical trials (Public Law 117-328). This legislative change formally recognized the scientific maturity of new approach methodologies (NAMs) and acknowledged the limited translational value of many animal studies for human-specific biologics.

### 4.2 New approach methodologies: an emerging paradigm for nonclinical assessment

New approach methodologies (NAMs) represent a potentially transformative paradigm in nonclinical pharmaceutical safety assessment, encompassing a diverse suite of scientific tools designed to evaluate drug safety and efficacy without relying solely on traditional animal testing. NAMs broadly include: (1) advanced *in vitro* systems, such as three-dimensional organoids, organ-on-chip (microphysiological) platforms, and human-derived primary cell and tissue models that recapitulate tissue-specific and organ-level human biology; (2) *in silico* computational approaches, including physiologically based pharmacokinetic (PBPK) modeling, quantitative systems pharmacology (QSP), artificial intelligence and machine learning-driven toxicity prediction, and receptor occupancy modeling; (3) in chemical assays for structural reactivity assessment; and (4) omics-based analyses (transcriptomics, proteomics, metabolomics) that provide mechanistic insights into drug-target interactions and off-target effects at the molecular level (Prior et al., 2020).

The FDA’s 2025 Roadmap to Reducing Animal Testing in Preclinical Safety Studies establishes a stepwise approach to integrating NAMs into regulatory decision-making, beginning with mAbs as the initial therapeutic class (FDA, 2025c). The roadmap envisions reducing routine 6-month non-human primate toxicology studies to 3 months when one-month studies and NAM-based assessments reveal no concerning safety signals, with the goal of making animal studies the exception rather than the norm for monospecific mAbs. Concurrently, the National Institutes of Health (NIH) announced in 2025 that it would no longer fund animal-exclusive studies, requiring all new proposals to include consideration for NAMs. For mAbs specifically, NAMs offer several potential advantages: human-relevant *in vitro* systems can model target engagement, cytokine release, and tissue cross-reactivity using human cells and tissues, thereby overcoming the species-specificity limitations that render many animal models non-informative for human-specific antibodies.

Limitations and challenges of NAM implementation. Despite the scientific promise of NAMs, significant challenges remain that warrant careful consideration. First, validation of NAMs for regulatory decision-making remains incomplete. While organ-on-chip platforms and three-dimensional organoid systems can model specific tissue-level responses, their ability to predict systemic toxicity across multiple organ systems simultaneously has not been fully demonstrated (Prior et al., 2020). Second, inter-laboratory reproducibility poses a substantial hurdle; standardized protocols for many NAM platforms are still under development, and variability in cell sources, culture conditions, and endpoint measurements across laboratories can lead to inconsistent results, complicating regulatory acceptance. Third, regulatory agencies have appropriately expressed skepticism about NAM sufficiency in certain high-risk contexts, particularly for first-in-class antibodies targeting novel pathways where prior clinical experience is limited and the consequences of missed toxicity signals could be severe. The EMA’s NAM qualification framework, while a positive step, acknowledges that fit-for-purpose qualification criteria must be established on a case-by-case basis, and that blanket acceptance of NAMs as replacements for animal studies remains premature for many product types. Fourth, global harmonization of NAM acceptance criteria poses additional challenges, as regulatory agencies across jurisdictions may apply different evidentiary standards for NAM qualification, potentially creating inconsistencies in development requirements across markets. Finally, the scalability of NAM platforms from academic proof-of-concept to routine regulatory use in industry settings requires further development of standardized manufacturing processes for biological components, validated reference standards, and commercially available platforms with demonstrated lot-to-lot consistency. These limitations do not negate the value of NAMs but underscore that their integration into regulatory decision-making should proceed incrementally, with rigorous validation at each step. A cautionary empirical example is the TGN1412 (theralizumab) case, in which conventional *in vitro* assays and preclinical animal models failed to predict the catastrophic cytokine release syndrome observed in first-in-human trials in 2006; subsequent analyses demonstrated that the cytokine storm was attributable to species-specific differences in CD28 expression density on effector memory T cells that neither standard *in vitro* systems nor non-human primate models adequately captured (Eastwood et al., 2010). While this event predates current NAM technologies, it illustrates the fundamental challenge of predicting complex immune-mediated toxicity and underscores why NAM qualification must include demonstrated sensitivity to rare but severe adverse events before animal studies can be comprehensively replaced (Table 2).

**TABLE 2:** Global regulatory positions on nonclinical safety testing for monoclonal antibodies.

Regulatory authority	Core guidance document	Nonclinical safety principle	Document status
FDA (United States)	Streamlined nonclinical safety studies for monospecific mAbs (2025)	Risk-based weight-of-evidence; animal studies only when uniquely informative; NAMs encouraged	Draft guidance (provisional)
EMA (European Union)	Biosimilar mAb Guideline (2012; updated 2022)	Avoid redundant *in vivo* studies; relevance-driven nonclinical assessment	Established guideline
WHO	Biosimilar guidelines (2022)	Stepwise approach; minimize non-informative animal testing	Established guideline
PMDA (Japan)	Biosimilar guidance (2020)	Case-by-case assessment based on pharmacological relevance	Established guideline
Health Canada	Biosimilar guidance (2016; updated 2021)	Comparative focus; avoid unnecessary nonclinical studies	Established guideline
ICH	S6(R1) biotechnology-derived pharmaceuticals (2011)	Pharmacologically relevant species; science-based study design	Established guideline

### 4.3 Scientific critique and regulatory evolution

The scientific necessity of animal testing for biosimilar antibodies has been questioned across multiple independent analyses. Van Meer et al. (2012) demonstrated that animal studies rarely predict serious post-marketing adverse events for biologics. Chapman et al. (2012) advocated for science-based reductions in animal testing for biopharmaceuticals. In a correspondence published in Science, Niazi (2022b) argued that animal studies rarely inform biosimilar regulatory decision-making and should be eliminated when analytical similarity and clinical pharmacology are sufficient to establish biosimilarity. Other perspectives, however, have noted that for certain product classes, particularly those with limited post-marketing safety databases or novel mechanisms, animal studies may continue to provide a degree of safety assurance that is difficult to replicate through other means (Bussiere et al., 2009; Sewell et al., 2014).

The FDA’s 2025 draft guidance on streamlined nonclinical safety studies for monospecific mAbs (FDA, 2025b)—which remains provisional pending finalization—indicates that long-duration non-rodent studies are generally unnecessary for monospecific mAbs and that comprehensive weight-of-evidence approaches may replace animal testing when no pharmacologically relevant species exists or when prior knowledge from the reference product and class is sufficient to characterize expected toxicities.

Table 2 summarizes the current regulatory positions on nonclinical safety testing expectations for mAbs across major regulatory authorities, distinguishing between established guidelines and emerging regulatory thinking reflected in draft documents.

## 5 Global convergence on nonclinical safety expectations for monoclonal antibodies

### 5.1 Alignment of regulatory science across jurisdictions

Over the past decade, major regulatory agencies have moved toward a shared scientific understanding that traditional nonclinical toxicology paradigms are frequently of limited relevance for mAbs and may constitute an inefficient use of development resources. This convergence reflects accumulated empirical evidence indicating that antibody-related toxicities are primarily predictable from target biology, tissue expression patterns, and pathway modulation rather than from nonspecific chemical liabilities or off-target effects characteristic of small-molecule drugs (Brennan et al., 2010; van Meer et al., 2012; Sewell et al., 2014).

**FIGURE 2:**
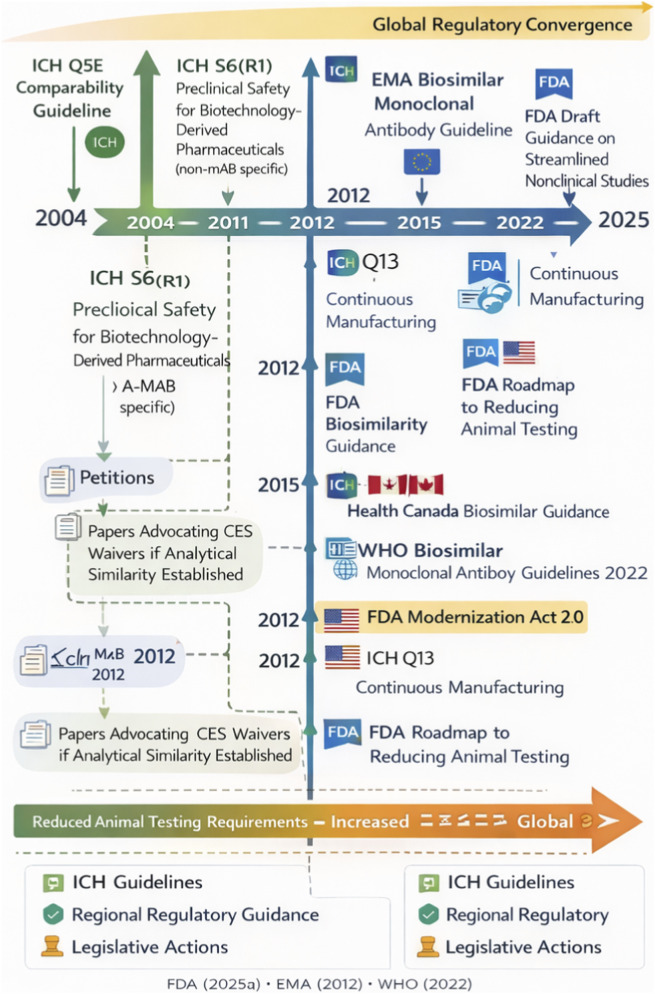
Timeline of Global Regulatory Evolution Toward Evidence-Efficient Antibody Development. This figure maps key regulatory milestones from EMA’s 2012 biosimilar mAb guideline through the FDA Modernization Act 2.0 (2022) and FDA’s 2025 draft guidance. Established regulatory requirements are distinguished from draft guidance proposals and emerging policy trends, consistent with the framework presented in Section 1.4. The timeline demonstrates the progressive convergence of major regulatory authorities toward evidence-efficient principles, while noting that draft positions (indicated separately) remain subject to finalization. Figures should be submitted in vector format (EPS or PDF) to ensure publication quality.

The EMA formalized this position early in its 2012 guideline on biosimilar mAbs, which states that nonclinical studies should be conducted only if they provide information not obtainable through analytical or functional comparison to the reference product (EMA, 2012). WHO’s 2022 biosimilar guideline adopted a similar stepwise, totality-of-evidence framework, explicitly cautioning against nonclinical studies that do not meaningfully reduce uncertainty or inform regulatory decision-making (WHO, 2022). PMDA has applied species relevance principles in practice, accepting biosimilar development programs with reduced or waived nonclinical studies when the reference product’s established safety profile and lack of pharmacologically relevant animal species support such approaches (Izutsu et al., 2016). However, it should be noted that the degree of convergence varies by jurisdiction, and practical implementation of these principles continues to differ across regulatory authorities (Figure 2).

### 5.2 Scientific limitations of animal models for antibody safety assessment

**FIGURE 3:**
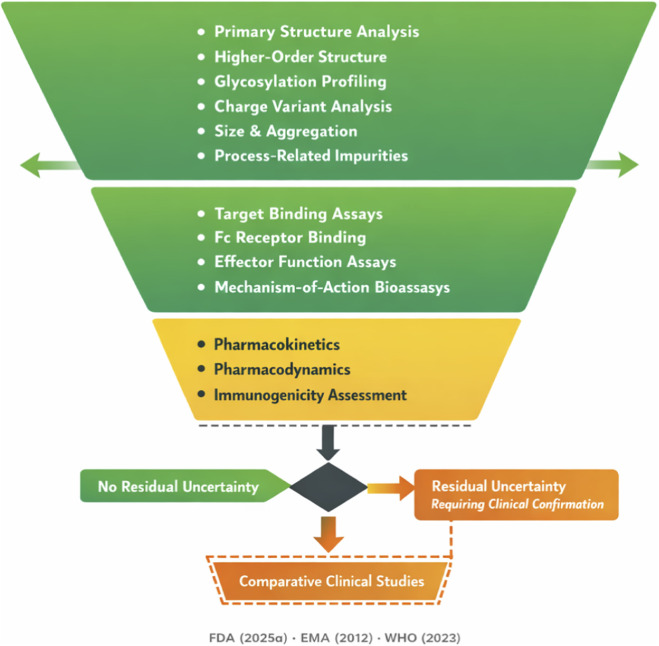
Conceptual Framework for Evidence-Efficient Biosimilar Antibody Development. This figure illustrates the proposed decision framework in which analytical characterization serves as the primary evidentiary foundation, with clinical pharmacology (PK/PD) studies and, where necessary, comparative clinical efficacy studies calibrated to residual uncertainty. The framework directly supports the central thesis of this review by demonstrating how each evidentiary tier addresses specific dimensions of uncertainty, and how the totality-of-evidence approach determines the extent of clinical confirmation required for individual biosimilar programs. Figures should be submitted in vector format (EPS or PDF) to ensure publication quality.

The regulatory shift away from routine animal testing is supported by research literature documenting the limited translational value of animal models for predicting human antibody safety. Non-human primates are often selected due to target cross-reactivity; however, immune-mediated toxicities, cytokine release patterns, and on-target effects observed in primates frequently fail to predict human outcomes with adequate sensitivity or specificity, particularly for antibodies targeting immune modulatory pathways where species differences are pronounced (Olson et al., 2000; van Meer et al., 2012; Grimaldi et al., 2016) (Figure 3).

Quantitative evidence on the predictive value of animal studies. Olson et al. (2000) reported that the overall concordance rate between animal toxicology findings and human adverse events for biologics was approximately 71% for non-human primates and 63% for rodent models; however, for immune-mediated and mechanism-based toxicities characteristic of mAbs, concordance rates were substantially lower. Van Meer et al. (2012) analyzed post-marketing safety data for 33 biopharmaceutical products approved in the EU and found that only 19% of serious post-marketing adverse events had been predicted by preclinical animal studies, with most unpredicted events related to immunogenicity and immune-mediated reactions—the very toxicities most relevant to mAb safety. Hay et al. (2014) further reported that approximately 57% of clinical trial failures for biologics were attributable to efficacy rather than safety, suggesting that animal toxicology studies, while detecting some safety signals, do not meaningfully reduce the risk of clinical failure for this product class. These data provide empirical support for the position that animal studies, while not without value, have demonstrable limitations in predicting the clinically relevant safety profile of therapeutic antibodies.

**FIGURE 4:**
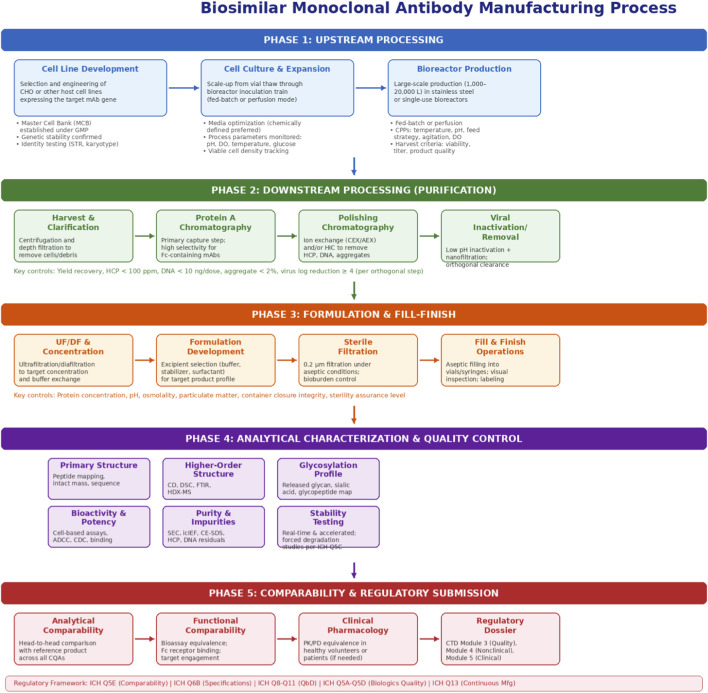
Biosimilar monoclonal antibody manufacturing process. The diagram illustrates the five principal phases: upstream processing (cell line development, cell culture, bioreactor production), downstream purification (harvest, Protein A chromatography, polishing, viral clearance), formulation and fill-finish, analytical characterization and quality control, and comparability with regulatory submission. Key process parameters, critical quality attributes, and applicable ICH guidelines are annotated at each stage. This figure supports the central thesis by demonstrating the multiple manufacturing control points at which analytical characterization ensures product consistency and comparability, reinforcing the premise that comprehensive process understanding underpins the analytics-first paradigm. Figures should be submitted in vector format (EPS or PDF) to ensure publication quality.

Comparative analyses of clinical trial failures further demonstrate that serious adverse events associated with therapeutic antibodies correlate more strongly with the mechanism of action and patient selection than with findings from animal toxicology (Kola and Landis, 2004; Hay et al. 2014). These data support the scientific rationale for supplementing animal studies with human-relevant NAMs, including *in vitro* target engagement assays, tissue cross-reactivity studies using human samples, receptor occupancy modeling, and mechanistic pharmacokinetic-pharmacodynamic simulations (Prior et al., 2020) (Figure 4).

## 6 Analytical similarity as the primary determinant of biosimilar development

### 6.1 Maturation of analytical science and orthogonal characterization

Analytical similarity assessment has become the dominant evidentiary pillar of biosimilar antibody development, reflecting both technological advancement and accumulated regulatory experience regarding the predictive power of comprehensive molecular characterization. Advances in mass spectrometry (including peptide mapping, intact mass analysis, and hydrogen-deuterium exchange), higher-order structure characterization (circular dichroism, Fourier-transform infrared spectroscopy, differential scanning calorimetry), and functional bioassays now allow a level of molecular resolution that far exceeds what was available during the early biosimilar era (Schiestl et al., 2011; Beck et al., 2013; Berkowitz et al., 2012).

Regulatory agencies consistently emphasize that the extent of residual uncertainty remaining after comprehensive analytical comparison determines the need for additional nonclinical or clinical data (FDA, 2015; EMA, 2012; WHO, 2022). The analytics-first paradigm, discussed by multiple regulatory science analyses (Schiestl et al., 2011; Weise et al., 2012; Kurki et al., 2017), holds that analytical similarity is not merely one element of the totality-of-evidence assessment but a central determinant from which other evidentiary requirements should logically follow.

Table 3 provides a comprehensive overview of the core analytical domains and representative methodologies used in the evaluation of biosimilar mAbs.

**TABLE 3:** Core analytical domains for biosimilar monoclonal antibody evaluation.

Analytical domain	Representative methods	Relevance to biosimilarity assessment
Primary structure	Peptide mapping (LC-MS/MS), intact mass analysis, disulfide bond mapping	Confirms amino acid sequence identity; detects PTMs and sequence variants
Higher-order structure	Circular dichroism, FTIR, DSC, HDX-MS	Ensures conformational similarity; detects folding differences
Glycosylation	Released N-glycan analysis, glycopeptide mapping, sialic acid quantification	Influences Fc effector functions and pharmacokinetics
Charge variants	icIEF, cation-exchange chromatography, capillary zone electrophoresis	Reflects chemical modifications; indicator of stability
Size variants	SEC, analytical ultracentrifugation, dynamic light scattering	Detects fragments and aggregates; relevant to immunogenicity
Target binding	SPR, bio-layer interferometry, isothermal titration calorimetry	Confirms equivalent antigen recognition
Fc receptor binding	SPR-based FcγR binding panels, FcRn binding assays, C1q binding	Predicts effector function and pharmacokinetic behavior
Functional activity	Cell-based potency assays, ADCC assays, CDC assays	Confirms molecular similarity translates to equivalent biological activity

### 6.2 Analytical sensitivity versus clinical efficacy endpoints

A growing body of evidence supports the regulatory position that analytical and functional assays are often more sensitive than clinical efficacy trials for detecting meaningful product differences. Clinical efficacy endpoints are influenced by disease heterogeneity, background therapies, patient selection criteria, inter-patient variability, and placebo effects, all of which can mask subtle but potentially relevant product differences (Schiestl et al., 2011; Kurki et al., 2017; Weise et al., 2012).

Important qualifications and counterexamples. While the analytical sensitivity argument is well supported for many biosimilar mAbs, several important qualifications merit discussion. First, there are documented instances where clinical endpoints detected immunogenicity differences that were not apparent from standard analytical panels. Anti-drug antibody (ADA) responses can differ between biosimilar and reference products due to factors such as host cell protein impurities, aggregation patterns, or formulation excipients that may fall within analytical similarity ranges but still provoke differential immune responses in patient populations (Schellekens, 2002; Doevendans and Schellekens, 2019). Doevendans and Schellekens (2019) provided an updated analysis of immunogenicity risk factors for biosimilar mAbs, emphasizing that aggregation, glycosylation differences, and formulation changes can influence ADA incidence in ways that may not be captured by standard analytical comparability panels. Such cases underscore that analytical similarity, while necessary, may not always be sufficient to predict immunogenicity outcomes. Second, pharmacokinetic equivalence has recognized limitations in the context of target-mediated drug disposition (TMDD). For antibodies whose clearance is substantially influenced by target-mediated pathways—such as those with high-affinity binding to cell-surface receptors at low doses—PK equivalence established in healthy volunteers may not fully reflect PK behavior in patient populations where target expression varies with disease state and severity (Mould and Meibohm, 2016). Third, the statistical frameworks used to establish equivalence margins in biosimilar studies have been subject to critique. Equivalence margins are derived from historical data on the reference product’s treatment effect, and the sensitivity of these margins to detect clinically meaningful differences depends on the assay sensitivity of the clinical model, the variability of the endpoint, and the sample size—all of which may introduce statistical fragility in certain therapeutic contexts (Christl et al., 2017). These considerations do not invalidate the analytics-first approach but highlight the importance of context-dependent application, with heightened scrutiny for products where immunogenicity, TMDD, or endpoint sensitivity may limit the predictive value of analytical and PK data alone.

## 7 Integration of analytical similarity with clinical pharmacology

### 7.1 Pharmacokinetic equivalence as the clinical bridge

Analytical similarity is integrated with clinical pharmacology to confirm that demonstrated molecular similarity translates into comparable *in vivo* behavior in humans. Pharmacokinetic (PK) studies are among the most sensitive clinical tools for detecting differences between antibody products, as pharmacokinetic parameters reflect the integrated outcome of multiple molecular attributes, including FcRn binding affinity, glycosylation patterns affecting clearance, aggregation propensity, target-mediated drug disposition, and immunogenicity (Wang et al., 2008; Mould and Meibohm, 2016).

Regulators increasingly prioritize PK equivalence studies—often conducted in healthy volunteers for antibodies without significant TMDD in the absence of disease—as the primary clinical confirmation of biosimilarity. When robust pharmacodynamic (PD) markers exist that are mechanistically linked to clinical outcome, combined PK/PD comparability may further reduce or eliminate the need for CES (EMA, 2012; FDA, 2015; WHO, 2022).

### 7.2 Pharmacodynamic markers and mechanism-based confirmation

For antibodies with validated pharmacodynamic biomarkers that correlate with clinical efficacy, PD endpoints can provide mechanism-based confirmation of comparable biological activity. Examples include reductions in absolute neutrophil count with anti-CD20 antibodies targeting B-cell depletion, receptor occupancy with checkpoint inhibitors, and cytokine suppression with anti-inflammatory antibodies (Kurki et al., 2017; Tesser et al., 2017).

A critical caveat, however, is that validated pharmacodynamic markers are not available for all therapeutic antibodies. For products targeting novel pathways or indications where biomarker-outcome relationships are not well established, PK equivalence alone may be insufficient to fully resolve residual uncertainty, and additional clinical confirmation may be warranted.

## 8 Scientific boundaries of biosimilarity: definability, reproducibility, and limitations of analytics-first approaches

### 8.1 Foundational requirements for biosimilarity assessment

The biosimilar regulatory paradigm is grounded in the fundamental ability to define and reproducibly demonstrate a multidimensional boundary of similarity around a reference product. This boundary is constructed by comprehensively characterizing multiple manufacturing lots of the reference product to establish its natural variability range and then demonstrating that the proposed biosimilar falls within justified similarity ranges across all critical quality attributes (Schiestl et al., 2011; Kurki et al., 2017; Weise et al., 2012).

Monoclonal antibodies produced via recombinant DNA technology are particularly amenable to this framework because they possess a single defined amino acid sequence encoded by cloned DNA, conserved immunoglobulin architecture with well-characterized domain structure, and extensively studied structure-function relationships accumulated across the therapeutic class.

### 8.2 Limitations and boundary conditions of analytics-first approaches

While the analytics-first paradigm represents a significant advance in evidence-efficient biosimilar development, it is essential to articulate the boundary conditions and limitations of this approach to avoid overgeneralization:

Heightened immunogenicity risk: For antibodies with complex immunogenicity profiles or reference products with known immunogenicity concerns, analytical similarity may not adequately predict immunogenicity across diverse patient populations. Factors such as aggregation propensity, host cell protein content, formulation differences, and patient immune status can influence immunogenicity in ways not fully captured by standard analytical panels. Extended immunogenicity surveillance and comparative immunogenicity studies may remain necessary for such products.

Novel targets with limited clinical experience: The predictive value of analytical similarity is strongest when structure-function relationships are well characterized through extensive prior clinical experience with the reference product and class. For antibodies targeting novel pathways or first-in-class mechanisms, the relationship between molecular attributes and clinical outcomes may be incompletely understood.

Insufficiently validated pharmacodynamic markers: The ability to waive CES is predicated on the availability of validated PD biomarkers that bridge between analytical/PK similarity and clinical efficacy. Where such biomarkers are unavailable, not validated, or where biomarker-outcome relationships are uncertain, the scientific basis for waiving CES is correspondingly weaker.

Complex mechanisms of action: Antibodies with multiple functional domains (e.g., bispecific or multispecific constructs) or those whose efficacy depends on the interplay among multiple mechanisms pose greater challenges for analytical similarity assessment, as more quality attributes become potentially critical.

Products with narrow therapeutic indices: For antibody-drug conjugates or other products where small differences in potency, distribution, or release kinetics could meaningfully affect the efficacy-toxicity balance, analytical similarity assessment must be correspondingly more stringent, and the threshold for additional clinical confirmation may be lower.

These limitations do not invalidate the analytics-first paradigm but rather define its appropriate scope of application. A scientifically rigorous approach requires explicit consideration of these boundary conditions in the design of biosimilar development programs.

## 9 Why polyclonal and non-monoclonal antibody products are not treated as biosimilars

Polyclonal antibody preparations—most notably plasma-derived hyperimmune globulins, anti-D immunoglobulins, and antivenom products—are intrinsically heterogeneous mixtures comprising thousands of distinct antibody species with differing antigen specificities, binding affinities, subclass distributions, and glycosylation profiles. Their molecular composition varies with donor population characteristics, vaccination or exposure history, plasma pool selection, and manufacturing conditions (Buchacher and Iberer, 2006; Lünemann et al., 2015).

WHO’s biosimilar guideline explicitly restricts its scope to biological products that can be ‘well characterized’ using state-of-the-art analytical methods and excludes plasma-derived products, vaccines, and other inherently heterogeneous preparations from the biosimilar pathway (WHO, 2022). This exclusion reflects a fundamental scientific constraint: without a stable, reproducible molecular fingerprint against which similarity can be assessed, head-to-head comparability cannot be meaningfully established.

The critical distinction is between measurable complexity (as in mAbs, where heterogeneity in glycosylation or charge variants can be quantified and controlled) and indeterminate complexity (as in polyclonal preparations, where the fundamental molecular composition cannot be fully defined). Only the former supports biosimilar conclusions.

## 10 Why conjugated antibodies May remain compatible with comparability-based regulation

### 10.1 Distinguishing measurable from indeterminate heterogeneity

Conjugated antibodies—including antibody-drug conjugates (ADCs), Fc-fusion proteins, radiolabeled antibodies, and other engineered constructs—are built on defined recombinant antibody backbones and incorporate additional functional moieties via covalent conjugation. Although conjugation introduces heterogeneity in attributes such as drug-to-antibody ratio (DAR), linker chemistry, payload distribution across conjugation sites, and stability of the linker-payload bond, these attributes are increasingly measurable and controllable using advanced analytical techniques (Beck et al., 2013; Wakankar et al., 2014; Bobaly et al., 2018).

### 10.2 Biosimilar ADCs: scientific complexity, not categorical exclusion

The absence of routine biosimilar ADC approvals in major jurisdictions to date reflects scientific and technical complexity rather than categorical regulatory exclusion. Demonstrating biosimilarity for ADCs requires alignment across multiple dimensions: the antibody backbone must meet conventional mAb similarity criteria, but additional demonstration of similarity is required for conjugation efficiency, drug loading and distribution, linker stability and catabolism kinetics, payload release profiles, and downstream catabolite characteristics (Wakankar et al., 2014; Bobaly et al., 2018).

Empirical evidence from post-approval manufacturing comparability exercises for trastuzumab emtansine (T-DM1) demonstrates that ADC comparability can be successfully established using comprehensive analytical approaches when process changes occur, providing a potential model for biosimilar development (Girish et al., 2012).

Table 4 contrasts the scientific characteristics of polyclonal antibodies and conjugated antibodies regarding their suitability for biosimilar development pathways.

**TABLE 4:** Polyclonal antibodies versus conjugated antibodies: Implications for biosimilarity assessment.

Attribute	Polyclonal antibodies	Conjugated antibodies (e.g., ADCs)
Molecular identity	Highly heterogeneous mixtures; thousands of distinct species	Defined recombinant backbone with characterized conjugation sites
Source of heterogeneity	Donor variability; immune response diversity	Conjugation efficiency; DAR distribution; linker chemistry
Analytical characterizability	Limited; complete characterization not feasible	Increasingly high; orthogonal methods available
Similarity boundary definability	Difficult or impossible; no stable reference	Potentially definable; requires expanded CQA set
Biosimilar regulatory pathway	Generally excluded; requires class-specific frameworks	Possible in principle; case-by-case evaluation

## 11 Waiver of comparative clinical efficacy studies: scientific and regulatory rationale

### 11.1 FDA’s 2025 draft guidance and its scientific foundation

In January 2025, the FDA issued draft guidance titled “Scientific Considerations in Demonstrating Biosimilarity to a Reference Product: Updated Recommendations for Assessing the Need for Comparative Efficacy Studies” (FDA, 2025a). This draft guidance—which remains provisional pending public comment and finalization—explicitly indicates that CES are not a default requirement for biosimilar approval and should be conducted only when residual uncertainty persists after comprehensive analytical, functional, and clinical pharmacology evaluations that cannot be resolved with available data.

Quantitative context for CES waivers. As of early 2025, the FDA has approved over 40 biosimilar products in the United States, the majority of which were approved following traditional development programs that included CES. However, the EMA has a longer track record with biosimilar mAbs, having approved more than 70 biosimilar products since 2006, including several mAb biosimilars in which reduced clinical requirements were accepted based on comprehensive analytical and PK data (Kurki et al., 2017; Wolff-Holz et al., 2019). Post-marketing pharmacovigilance data from these European approvals, as analyzed by Wolff-Holz et al. (2019) across more than a decade of biosimilar use, encompassing millions of patient exposures, have not identified safety or efficacy signals that would have been detected by CES but were missed by analytical and PK characterization, providing empirical support for the sufficiency of evidence-efficient approaches in this product class. It should be noted, however, that the absence of detected signals does not definitively prove that CES provides no additional value, as pharmacovigilance systems have inherent sensitivity limitations and the cumulative patient-years of exposure for individual biosimilar mAbs vary considerably.

The FDA’s evolving position reflects growing recognition that clinical efficacy trials are often statistically underpowered and biologically insensitive to detecting small differences in quality between biosimilar and reference products (Christl et al., 2017). Multiple independent analyses have supported this position, with Schiestl et al. (2011), Weise et al. (2012), and Kurki et al. (2017) providing evidence that analytical and functional assays often exhibit greater discriminatory power than clinical endpoints.

It is important to note that some regulatory science analyses, including those by Niazi (2022a); Niazi (2025a), have advocated for broader waivers of CES requirements. The FDA’s draft guidance takes a more nuanced approach, retaining CES as a potential requirement when residual uncertainty cannot be resolved through other means; its final position will be established after public comment and agency review.

### 11.2 Alignment with EMA, WHO, and international regulatory practice

EMA’s biosimilar mAb guideline historically stated that similar clinical efficacy should ‘normally’ be demonstrated, but the guideline also emphasized the importance of sensitive clinical models and acknowledged circumstances in which pharmacodynamic endpoints may suffice (EMA, 2012). EMA’s practice has evolved toward an increasingly flexible application of this principle, with several biosimilar approvals based on comprehensive analytical and PK data (Kurki et al., 2017; Wolff-Holz et al., 2019).

WHO’s 2022 biosimilar guidelines reinforce a flexible, stepwise approach in which the extent of clinical data required depends explicitly on the degree of residual uncertainty remaining after analytical and functional characterization (WHO, 2022). The direction toward evidence-efficient CES requirements across these major regulatory authorities suggests growing international alignment, though implementation details continue to vary across jurisdictions, and the pace of harmonization remains uneven.

Table 5 identifies product categories that are less amenable to routine waiver of CES.

**TABLE 5:** Product types less amenable to routine CES waiver and underlying scientific rationale.

Product type	Scientific rationale	Potential path forward
Polyclonal antibodies	Outside biosimilar scope; indeterminate heterogeneity	Product-class-specific frameworks
Vaccines	Excluded from biosimilar frameworks; complex immunological responses	Dedicated vaccine regulatory pathways
Antibody-drug conjugates	Multi-attribute complexity; limited regulatory precedent	Expanded analytical characterization; case-by-case evaluation
Bispecific/multispecific antibodies	Complex mechanism with multiple binding interactions	Comprehensive functional panels; evolving guidance
Products lacking sensitive PD markers	Residual uncertainty not resolvable through PK alone	Development of validated biomarkers
Products with complex immunogenicity	Long-term effects not predictable from short-term PK	Extended immunogenicity surveillance

## 12 Manufacturing, comparability, and lifecycle management of antibody products

### 12.1 Comparability as a continuous lifecycle obligation

Manufacturing changes are inevitable across the lifecycle of biologic products, including mAbs and their biosimilars. Changes in cell substrates, culture media composition, raw materials, manufacturing scale, production site, purification processes, or formulation parameters are routinely introduced (Lubiniecki, 2016; FDA, 2015). Recognizing this reality, regulators have long emphasized comparability—rather than re-demonstration of clinical efficacy—as the scientifically appropriate framework for managing post-approval manufacturing changes.

ICH Q5E establishes the global standard for demonstrating that manufacturing changes do not adversely affect product quality, safety, or efficacy of biotechnology-derived products (ICH, 2004). The comparability exercise relies primarily on analytical and functional assessment to demonstrate that pre- and post-change products remain within justified similarity ranges across critical quality attributes. This paradigm is conceptually similar to biosimilar development: both frameworks rely on analytical similarity as the primary determinant of clinical continuity.

This conceptual symmetry between post-approval comparability and biosimilar development has been noted by multiple regulatory science analyses (Weise et al., 2012; Wolff-Holz et al., 2019). If analytical comparability is sufficient to manage manufacturing changes to originator products without new clinical efficacy trials, the scientific rationale for requiring such trials for biosimilars that demonstrate equivalent analytical similarity warrants careful consideration. However, it should be acknowledged that the regulatory context differs; originator-comparability exercises benefit from the originator product’s accumulated clinical experience database, which may reduce the perceived risk of an analytical-only assessment.

### 12.2 Current regulatory guidelines governing mAb manufacturing and development

The manufacturing and development of mAbs is governed by a comprehensive, internationally harmonized regulatory framework encompassing cell line development, upstream and downstream processing, analytical characterization, quality control, and lifecycle management. At the international level, ICH has established foundational quality guidelines, including: ICH Q5A(R1) on viral safety evaluation; ICH Q5B on analysis of the expression construct; ICH Q5C on stability testing of biotechnological products; ICH Q5D on derivation and characterization of cell substrates; and ICH Q5E on comparability of biotechnological products subject to manufacturing process changes.

The quality-by-design (QbD) framework, formalized through ICH Q8(R2) (Pharmaceutical Development), ICH Q9(R1) (Quality Risk Management), ICH Q10 (Pharmaceutical Quality System), and ICH Q11 (Development and Manufacture of Drug Substances), provides a systematic, science- and risk-based approach to manufacturing process development. Under QbD principles, manufacturers define a target product quality profile (QTPP), identify critical quality attributes (CQAs) through risk assessment, establish functional relationships between process parameters and CQAs, and define a design space within which the process can operate while maintaining product quality. ICH Q12 (Lifecycle Management) further extends these principles by establishing a framework for managing post-approval changes to chemistry, manufacturing, and controls (CMC).

At the regional level, the FDA’s regulatory framework includes guidance on process validation outlining a lifecycle approach encompassing process design, process qualification, and continued process verification. The EMA’s Guideline on Development, Production, Characterisation and Specifications for Monoclonal Antibodies provides a comprehensive framework addressing manufacturing process development, characterization, specification setting, and reference standard establishment. WHO’s Guidelines for the Safe Production and Quality Control of Monoclonal Antibodies provide similar manufacturing guidance with relevance to global harmonization and prequalification of products for low- and middle-income countries.

### 12.3 Continuous manufacturing and enhanced process control

ICH Q13, finalized in 2022, extends comparability principles by formally recognizing continuous manufacturing as an acceptable paradigm for drug substances and drug products, including biologics (ICH, 2022). Continuous manufacturing offers potential advantages in process control, product consistency, manufacturing efficiency, and supply chain responsiveness (Lee et al., 2015; Konstantinov and Cooney, 2015).

## 13 An integrated future development model for innovator and biosimilar antibodies

By synthesizing regulatory guidance, peer-reviewed research from multiple independent groups, and accumulated regulatory experience, a potential future development model for mAbs can be considered—one that, if current draft positions are finalized and adopted across jurisdictions, would apply rationalized evidentiary principles across both the innovator and biosimilar pathways. The realization of such a model remains subject to regulatory discretion in each jurisdiction and to the outcome of ongoing public consultations.

For innovator mAbs, if current regulatory trends continue, development may increasingly emphasize early mechanistic understanding, target biology characterization, human-relevant nonclinical methods including NAMs, and model-informed clinical pharmacology approaches. In jurisdictions adopting similar reforms, extended animal toxicology programs could be limited to circumstances in which they provide unique, decision-relevant information not obtainable through other means (Prior et al., 2020).

For biosimilar antibodies, if draft guidance positions are finalized as proposed, development would continue to be anchored in totality-of-evidence approaches. Comprehensive analytical and functional characterization would serve as the foundation, with PK equivalence studies serving as the primary clinical bridge between analytical similarity and clinical practice. CES would be conducted only when residual uncertainty remains after all other evidence has been evaluated. However, the extent to which individual regulatory authorities adopt these streamlined approaches may vary, and sponsors should anticipate jurisdiction-specific requirements during the transition period.

These developments, taken together with the FDA Modernization Act 2.0 and the adoption of analytics-first principles by EMA, WHO, and other authorities, suggest a possible direction toward evidence-efficient development. The final scope and uniformity of implementation, however, remain subject to ongoing regulatory deliberations across jurisdictions (see Section 1.4 for the framework distinguishing established requirements from draft proposals).

## 14 Implications for global access, cost, and healthcare sustainability

The transition toward evidence-based antibody development has far-reaching implications for global health, healthcare economics, and the sustainability of access to biologic medicines. Large-animal programs and CES impose substantial costs and time burdens, particularly for biosimilar developers operating in resource-constrained settings (Mulcahy et al., 2018; Moorkens et al., 2021). Reducing or eliminating non-informative studies can lower development costs, shorten timelines to market approval, and increase the commercial viability of biosimilar programs.

WHO has emphasized that modernized, science-based biosimilar frameworks are essential for improving access to biologic therapies in low- and middle-income countries (WHO, 2022; Moorkens et al., 2017). Regulatory convergence around analytics-first principles facilitates international reliance pathways, reduces duplicative regulatory requirements across jurisdictions, and enables more rapid global diffusion of biosimilar products.

However, it must be acknowledged that streamlining development pathways also requires robust regulatory infrastructure to ensure that analytical comparability assessments are conducted with sufficient rigor and that post-marketing surveillance systems can detect unexpected safety signals—investments that may themselves present challenges in resource-limited settings.

Table 6 summarizes the economic, access, and sustainability implications of evidence-efficient biosimilar development.

**TABLE 6:** Comparative impact of evidence-efficient versus traditional biosimilar development paradigms.

Development dimension	Traditional paradigm	Evidence-efficient paradigm
Development timeline	7–10 years	4–6 years with streamlined requirements
Development cost	$100–250 million per program	$50–100 million with reduced clinical requirements
Barriers to market entry	High; limits competition	Lower; enables broader developer participation
Price competition	Delayed; modest price reductions	Earlier; meaningful reductions (30%–80%)
Global access	Limited LMIC penetration	Broader availability; affordable pricing
Ethical considerations	Continued animal testing; patient exposure in non-informative trials	3Rs-aligned; scientifically necessary studies only
Safety assurance	No demonstrated safety advantage	Equivalent assurance through analytical/PK characterization

## 15 Conclusion

The development of therapeutic and biosimilar mAbs is being reshaped by a convergence of scientific maturity, technological advancement, and regulatory evolution toward evidence-efficient paradigms. Advances in analytical science—including mass spectrometry, higher-order structure characterization, and comprehensive functional bioassay panels—provide molecular resolution that enables robust demonstration of biosimilarity at the level of critical quality attributes. A deeper mechanistic understanding of antibody biology, target pharmacology, and structure-function relationships supports science-based risk assessment.

Regulatory agencies worldwide now explicitly recognize mAbs as a distinct and favorable class of therapeutic proteins for evidence-efficient development, reflecting their shared immunoglobulin scaffold, predictable pharmacokinetics, mechanism-based safety profiles, and extensive accumulated regulatory experience. The FDA’s 2025 draft guidance on streamlined nonclinical studies and conditional clinical efficacy requirements, while remaining subject to finalization following public comment, represent current regulatory scientific thinking and signal the direction of future policy.

At the same time, clear scientific boundaries remain that appropriately constrain the scope of streamlined development. Polyclonal antibody preparations fall outside the biosimilar paradigm because intrinsic, indeterminate heterogeneity precludes the definable similarity boundaries upon which comparability assessment depends. Conjugated antibodies and multispecific constructs introduce additional complexity, though they may remain compatible with comparability-based development when that complexity is measurable and controllable. Analytics-first approaches have recognized limitations in scenarios involving heightened immunogenicity risk, novel targets with limited clinical experience, insufficiently validated pharmacodynamic markers, or products with narrow therapeutic indices. NAMs, while promising, require further validation, standardization, and regulatory qualification before they can comprehensively replace traditional nonclinical approaches across all product types and regulatory jurisdictions.

The trajectory of regulatory reform, supported by independent regulatory science scholarship, formal regulatory deliberations, and accumulated clinical experience, points toward antibody development that is more efficient, more ethical in its approach to animal use and patient exposure, and more globally accessible. However, the realization of this vision depends on continued investment in analytical science validation, NAM development and standardization, robust post-marketing surveillance infrastructure, and ongoing dialogue among regulators, industry, and academia to ensure that streamlining does not compromise the safety, efficacy, or quality standards essential to public health protection.

It is important to acknowledge several sources of uncertainty that may affect the pace and scope of regulatory reform. First, global harmonization timelines remain unpredictable; while the FDA, EMA, and WHO have signaled directional alignment, smaller regulatory authorities and emerging markets may adopt different timelines or maintain more conservative evidentiary requirements, creating a patchwork of regulatory expectations that sponsors must navigate. Second, some regulatory scientists and industry stakeholders maintain legitimate concerns that premature elimination of clinical efficacy studies could erode the safety net for products with unanticipated immunogenicity or subtle efficacy differences detectable only in patient populations (Christl et al., 2017). Third, political, legal, and institutional factors—including changes in regulatory leadership, legislative priorities, and the outcomes of public comment periods—may alter the trajectory of draft guidance finalization in ways that are difficult to predict. The author acknowledges these uncertainties and emphasizes that the analytics-first paradigm should be implemented incrementally, with continued pharmacovigilance serving as an essential complement to streamlined pre-approval requirements.
